# Supplementary material for: Exploration of Antimicrobial Ingredients in Psoralea corylifolia L. Seed and Related Mechanism against Methicillin-Resistant Staphylococcus aureus
Source: Molecules. 2022 Oct 17;27(20):6952. doi: 10.3390/molecules27206952 (PMC9611313; doi:10.3390/molecules27206952)
Supplement: Supplementary file 1 [file molecules-27-06952-s001.zip › molecules-1956077-supplementary.pdf]

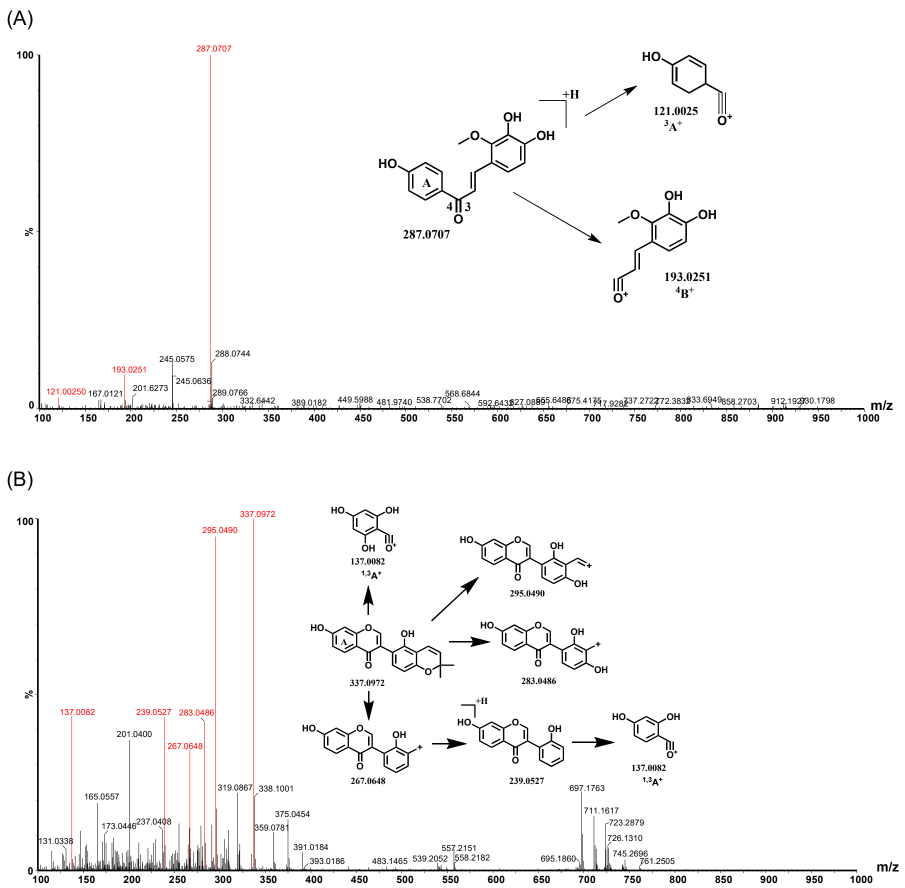

Figure S1. Typical fragments ions and the fragmentation pathwayof reference flavonoids in PI mode. (**A**) licochalcone D and (**B**) glabrone.

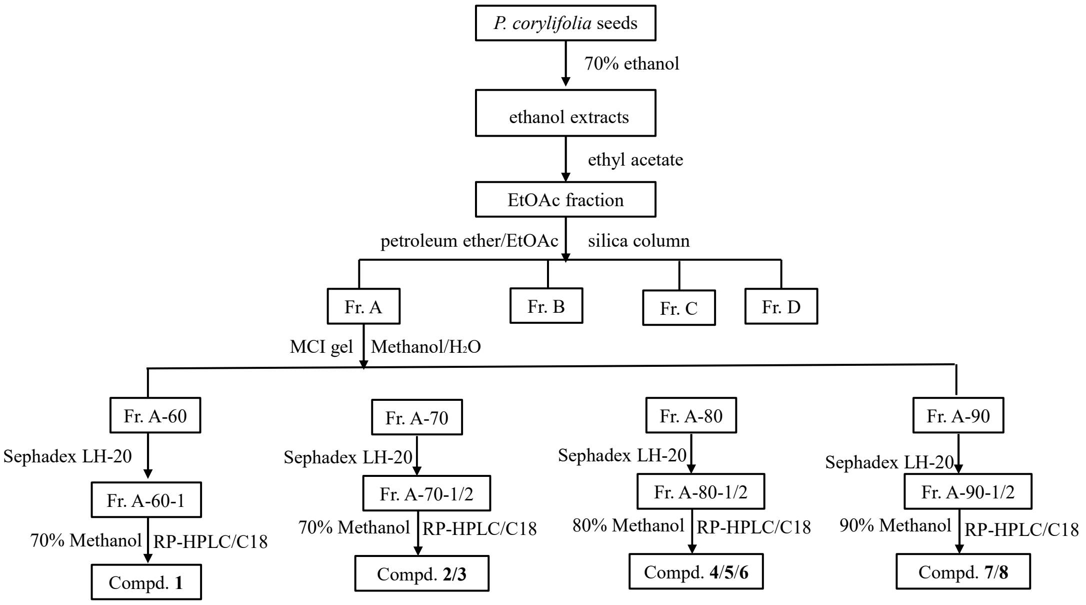

Figure S2. Separation process of Fr. A–D and compounds **1-8** from PCS.
